# Machine learning-based integration reveals reliable biomarkers and potential mechanisms of NASH progression to fibrosis

**DOI:** 10.1038/s41598-025-97670-4

**Published:** 2025-04-11

**Authors:** Jiahui Feng, Zheng Gong, Jialing Yang, Yuting Mo, Fengqian Song

**Affiliations:** 1 Department of Gastroenterology, Loudi Central Hospital, Loudi, Hunan China; 2 Department of Pharmacy, Union Hospital, Tongji Medical College, Huazhong University of Science and Technology, Wuhan, China; 3 School of Basic Medical Sciences, Nanjing Medical University, Nanjing, Jiangsu China; 4 Department of Geriatrics, Zhongnan Hospital of Wuhan University, Wuhan, China

**Keywords:** Machine learning, NASH, Fibrosis, Biomarkers, Immune microenvironment, Machine learning, Hepatology, Liver diseases

## Abstract

Non-alcoholic fatty liver disease (NAFLD) affects about 25% of adults worldwide. Its advanced form, non-alcoholic steatohepatitis (NASH), is a major cause of liver fibrosis, but there are no non-invasive tests for diagnosing or preventing it. In our study, we analyzed data from multiple sources to find crucial genes linked to NASH fibrosis. We built diagnostic models using 103 machine learning algorithms and validated them with two external datasets. All models performed well, with the best one (RF + Enet[alpha = 0.6]) achieving an average AUC of 0.822. This model used five key genes: LUM, COL1A2, THBS2, COL5A2, and NTS. Our findings show that these genes are important in collagen and extracellular matrix pathways, shedding light on how NASH progresses to liver fibrosis. We also found that certain immune cells, like M1 macrophages, are involved in this process. This study provides a reliable diagnostic tool for assessing fibrosis risk in NASH patients and suggests potential for immunotherapy, laying a foundation for future treatments.

**Supplementary Information:**

The online version contains supplementary material available at 10.1038/s41598-025-97670-4.

## Introduction

Considering that nearly 40% of the global population is overweight or obese, non-alcoholic fatty liver disease (NAFLD) has swiftly emerged as a major health concern, currently affecting about 25% of adults worldwide^1,2^. Liver fibrosis develops as a consequence of chronic inflammation, typically after prolonged periods of sustained or recurrent tissue injury and immune response. This condition is marked by the accumulation of extracellular matrix (ECM) components, leading to structural alterations in the liver, particularly through the deposition of collagen and other fibrous proteins like elastin in the Disse space. Excessive ECM production can disrupt liver architecture, compromise organ function, and impair intrahepatic blood flow, eventually progressing to cirrhosis. While the incidence of liver fibrosis associated with hepatitis B and C has declined due to advances in treatment and vaccination programs, fibrosis resulting from non-alcoholic steatohepatitis (NASH) is on the rise, becoming a leading cause of liver transplantation^3^. Despite the substantial burden posed by NASH-related liver diseases, there are currently no approved therapies specifically targeting NASH^4^. Thus, identifying key genes involved in the progression of NASH to liver fibrosis is essential for discovering reliable therapeutic targets to reverse this condition.

One of the significant clinical challenges is the lack of accurate tools to predict the risk of liver fibrosis in NASH patients who do not yet have fibrosis, even though methods like the NAS score provide reliable assessments of NASH severity^5^. This limitation hampers the ability to determine appropriate therapeutic interventions. While histological evaluation remains the gold standard for diagnosing, prognosticating, and monitoring NAFLD, it is constrained by variability in accuracy and reliance on pathologists’ expertise^6^. Developing a simple, reproducible, and non-invasive method to assess the risk of liver fibrosis in NASH patients would allow for targeted treatment of high-risk individuals, potentially reducing the incidence of end-stage liver disease. Advances in artificial intelligence, particularly in machine learning, offer a more efficient approach to knowledge discovery and the creation of predictive models with greater accuracy than traditional statistical methods. Machine learning is particularly advantageous in analyzing large, high-dimensional clinical datasets^7^. Recent studies have successfully employed bioinformatics and machine learning techniques to identify potential feature genes for NAFLD, with promising validation outcomes^7,8^. Therefore, constructing a risk model for NASH sfibrosis using machine learning is not only feasible but also holds significant clinical promise.

## Materials and methods

### Data retrieval and collection

We used keywords such as “NAFLD”, “NASH”, “Fatty liver” to search, all the datasets related to NASH and NASH fibrosis and contains clear information on the distinction between NASH and NASH fibrosis (such as NAS score) were obtained from the Gene Expression Omnibus (GEO) database, including GSE48452, GSE49541, GSE89632, GSE130970, and GSE163211 (https://www.ncbi.nlm.nih.gov/geo/). We downloaded gene expression profile data, relevant annotation files, and clinical information, including fibrosis staging, for all datasets. To maintain a minimal discrepancy between the sizes of the training and validation sets and include as many datasets as possible in the training set, thereby preventing model overfitting and enhancing the reliability of the model during validation (avoiding selection bias due to an excess of training samples and a paucity of validation samples), the datasets GSE48452, GSE49541, and GSE89632 were assigned as training datasets, comprising a total of 48 samples with non-fibrotic NASH and 61 samples with fibrotic NASH. Specifically, GSE48452 includes 4 samples with NASH without fibrosis and 14 samples with NASH fibrosis; GSE49541 comprises 40 samples with NASH without fibrosis and 32 samples with NASH fibrosis; GSE89632 includes 4 samples with NASH without fibrosis and 15 samples with NASH fibrosis. The datasets GSE130970 and GSE163211 served as validation datasets. GSE130970 contains 11 samples with NASH without fibrosis and 49 samples with NASH fibrosis, while GSE163211 consists of 72 samples with NASH without fibrosis and 82 samples with NASH fibrosis.

### Data normalization and differential analysis

For RNA-seq data analysis, quantile normalization of gene expression was conducted using the normalizeBetweenArrays function from the limma package, followed by log2 transformation. To visualize relative gene expression between NASH non-fibrotic and fibrotic tissues, the ggplot2 package was utilized. Differentially expressed genes (DEGs) associated with NASH fibrosis were identified using the limma package, applying criteria of |FC| ≥ 1.5 and FDR < 0.05^9^.

### Weighted gene co-expression network analysis (WGCNA)

WGCNA is a powerful tool for identifying gene networks, co-expressed gene modules, and pivotal genes linked to phenotypic traits. In this study, we integrated training datasets to identify gene modules that are significantly associated with NASH fibrosis using the WGCNA R package. To evaluate the network’s scale-free topology, we tested various β values and selected β = 4 based on the scale-free topology criteria. Genes with similar expression patterns were then grouped into modules using the “dynamic tree cut” algorithm. Additionally, Pearson correlation analysis was conducted to explore the relationship between module genes and clinical traits^10^.

### Gene enrichment functional analysis

To uncover the molecular mechanisms underlying fibrosis in NASH, we conducted a comprehensive gene ontology (GO) analysis, covering GO biological processes (GO BP), GO cellular components (GO CC), and GO molecular functions (GO MF), along with KEGG pathway analysis using the clusterProfiler R package. The top 30 enriched terms were visualized using bubble plots to provide a clear representation of the enrichment results.

In addition, Gene Set Enrichment Analysis (GSEA) was utilized to detect potential changes in biological functions between different NASH subgroups^11^. The significance criteria were set at |normalized enrichment score (NES)| > 1 and a nominal *p*-value < 0.05. These analyses revealed key differentially expressed genes and enriched pathways/processes.

For Gene Set Variation Analysis (GSVA), we downloaded two gene sets, “c2.cp.kegg.v7.4.symbols” and “c5.go.bp.v7.5.1.symbol,” from the MSigDB database (https://www.gsea-msigdb.org/gsea/msigdb/)^12^. Using the limma package, we calculated GSVA scores to evaluate the differential enrichment of functions and pathways across ERS-related subtypes. Pathways with a |t-value| > 2 were considered significantly enriched.

### Construction and validation of a diagnostic model for NASH fibrosis

The study focused on binary classification tasks by evaluating 12 distinct machine learning algorithms. Regression-based approaches, including Elastic Net (Enet), Ridge, Stepwise Generalized Linear Model (Stepglm), and Least Absolute Shrinkage and Selection Operator (LASSO), were included due to their strengths in handling high-dimensional data and feature selection, which helps mitigate overfitting. Additionally, classification algorithms such as Support Vector Machines (SVM), Linear Discriminant Analysis (LDA), glmBoost, Partial Least Squares Regression Generalized Linear Model (plsRglm), Random Forest, Gradient Boosting Machine (GBM), Extreme Gradient Boosting (XGBoost), and Naive Bayes were implemented. A total of 103 models were generated by combining these algorithms.To prevent overfitting, two strategies were employed: first, cross-validation was performed, where one algorithm handled variable selection, and another built the classification model; second, the validation dataset size was increased. The area under the receiver operating characteristic (ROC) curve was calculated for all 103 models using the training set, and a heatmap was used to visualize the results, identifying the best-performing algorithms. The genes selected by the top-performing algorithm were subsequently used to compute ROC values for diagnosing NASH fibrosis in both the training and validation datasets.

### Evaluation of immune infiltration patterns in NASH fibrosis

The CIBERSORT algorithm was employed to quantify the infiltration of 22 immune cell types across NASH subgroups^13^. Group differences were evaluated using the Student’s t-test and visualized using the ggboxplot R package. Correlations between the model genes and immune cell types were analyzed with the corrplot package and displayed using pheatmap.

### Statistical analysis

All statistical analyses were conducted using R software (version 4.3.1, https://www.r-project.org) along with the relevant R packages^13^. Data are presented as mean ± standard error (SE). The t-test was used for comparisons between two groups, while one-way ANOVA was employed for comparisons involving three or more groups. Spearman’s correlation analysis was performed using the ggpubr and stats packages. Statistical significance was defined as *p*-values < 0.05. Significance levels are denoted as follows: * (*p* < 0.05), ** (*p* < 0.01), *** (*p* < 0.001).

To account for multiple testing in differential gene expression and pathway enrichment analyses, the false discovery rate (FDR) was controlled using the Benjamini-Hochberg method. Genes and pathways with FDR-adjusted p-values below 0.05 were deemed statistically significant. Additionally, during machine learning-based model construction, stringent feature selection criteria were applied to prevent overfitting.

## Results

### Identification of differentially expressed genes in NASH fibrosis

**Fig. 1:**
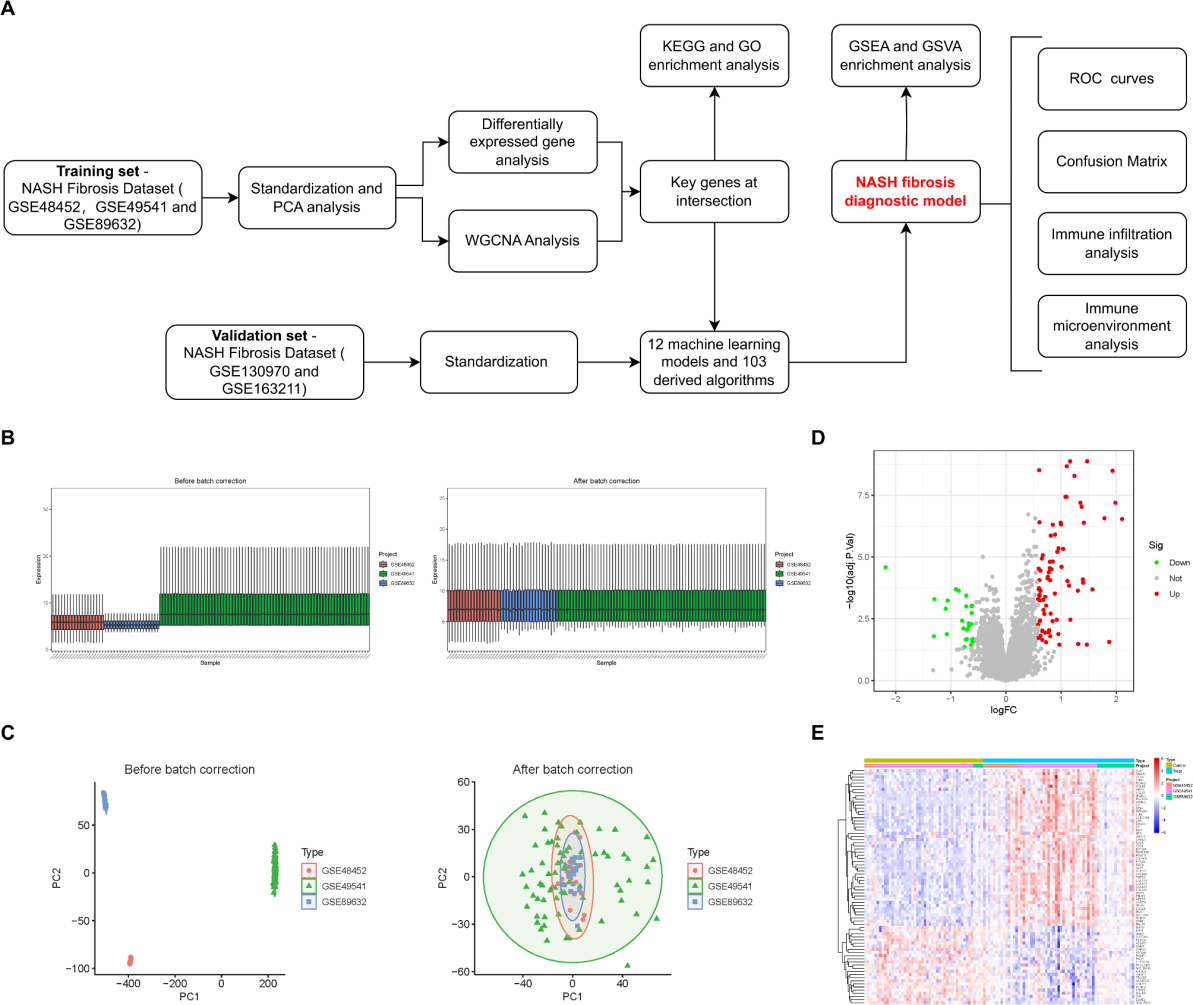
Standardization and differential analysis of NASH data. (**A**) Detailed flowchart illustrating our screening and validation process. (**B**) Box plot demonstrating data integration before and after batch effect correction. (**C**) PCA (Principal Component Analysis) analysis showing sample distribution before and after batch effect correction. (**D**) Volcano plot highlighting differentially expressed genes in NASH fibrosis. (**E**) Heatmap displaying the top 50 genes with the highest differential expression fold change in NASH fibrosis. All images were created using R software (version 4.3.1, https://www.r-project.org). Statistical significance is denoted as *p < 0.05, **p < 0.01, ***p < 0.001.

Our study proceeded according to the flowchart depicted in Fig. 1A. We combined the GSE48452, GSE49541, and GSE89632 datasets, all of which included data related to NASH fibrosis staging. Box plots demonstrated that our normalization technique effectively minimized batch effects across the datasets (Fig. 1B). Principal Component Analysis (PCA) further confirmed that, after batch effect correction, the datasets GSE48452, GSE49541, and GSE89632 displayed consistent distributions (Fig. 1C), ensuring the quality of the integrated dataset. Next, to pinpoint genes with abnormal expression in NASH fibrosis, we identified 104 differentially expressed genes (DEGs), consisting of 25 upregulated and 79 downregulated genes, when comparing the NASH fibrosis group to the non-fibrosis group (Fig. 1D, Table S1). Additionally, we created a heatmap to visualize the expression levels of the top 50 genes ranked by log fold change (logFC) in NASH across various groups (Fig. 1E).

### Identification and functional enrichment analysis of key gene modules in NASH fibrosis

**Fig. 2:**
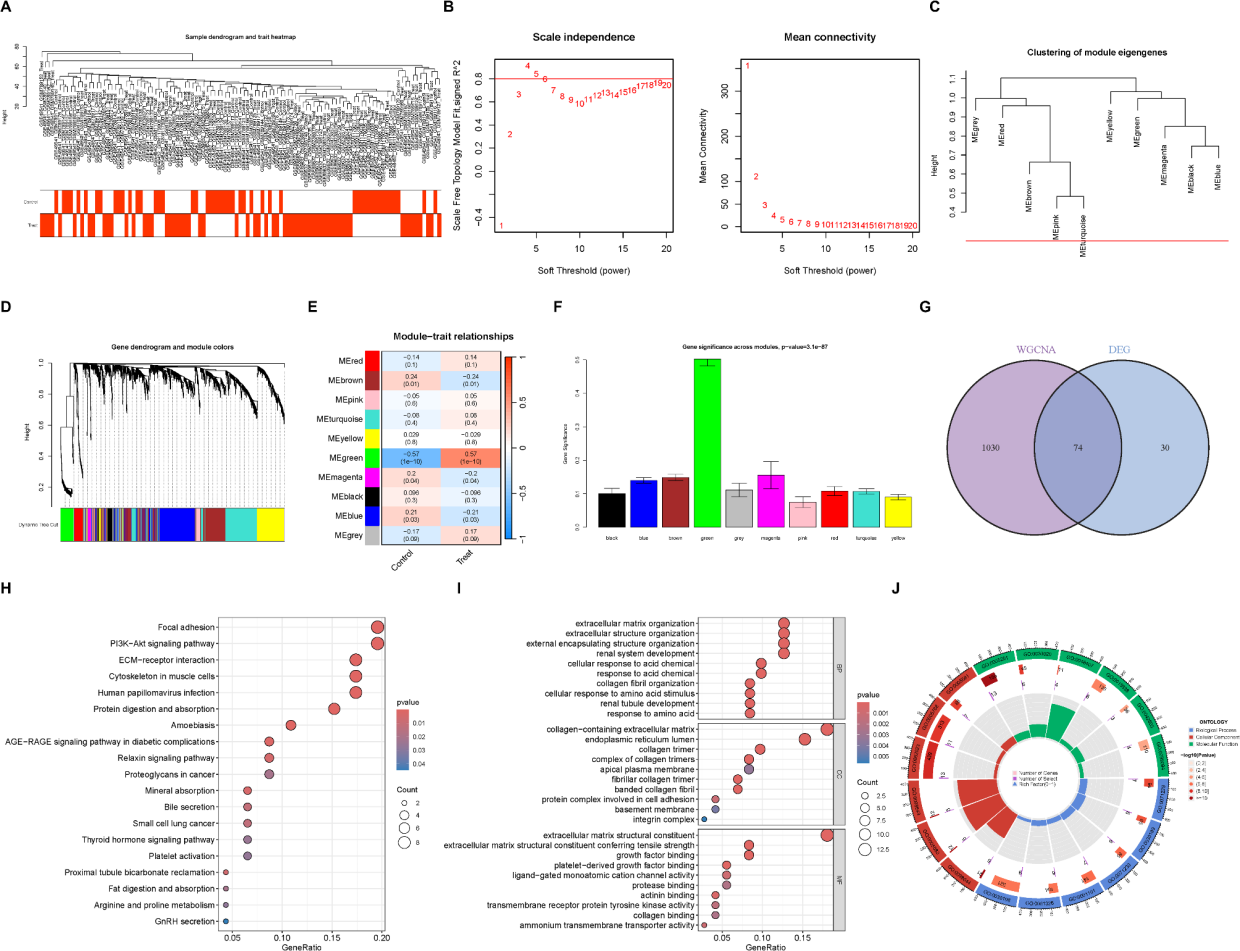
WGCNA and functional enrichment analysis. (**A**) Analysis of sample clustering and identification of outliers. (**B**) Scale-free fit index analysis across different soft threshold powers (β). (**C**) Dendrogram cut at a height of 0.25 to detect and merge similar modules. (**D**) Original and merged modules under the dendrogram. (**E**) Heatmap of module-trait relationships, with each cell displaying the corresponding correlation and p-value. (**F**) Bar graph showing gene significance across different modules. (**G**) Venn diagram illustrating the intersection between differentially expressed genes and key gene modules identified by WGCNA. (**H**) Bubble chart displaying the KEGG enrichment analysis results for the intersected gene set. (**I**) Bubble chart presenting the GO enrichment analysis results for the intersected gene set. (**J**) Circular plot showing the GO enrichment analysis results for the intersected gene set, including annotations for each enriched pathway. GO:0071229: cellular response to acid chemical; GO:0030199: collagen fibril organization; GO:0071230: cellular response to amino acid stimulus; GO:0001101: response to acid chemical; GO:0061326: renal tubule development; GO:0030198: extracellular matrix organization; GO:0098644: complex of collagen trimers; GO:0005583: fibrillar collagen trimer; GO:0098643: banded collagen fibril; GO:0062023: collagen-containing extracellular matrix; GO:0005788: endoplasmic reticulum lumen; GO:0005581: collagen trimer; GO:0005201: extracellular matrix structural constituent; GO:0030020: extracellular matrix structural constituent conferring tensile strength; GO:0048407: platelet-derived growth factor binding; GO:0019838: growth factor binding; GO:0042805: actinin binding; GO:0099094: ligand-gated monoatomic cation channel activity. All images were created using R software (version 4.3.1, https://www.r-project.org). Statistical significance is denoted as *p < 0.05, **p < 0.01, ***p < 0.001.

Clustering analysis was conducted on NASH samples using R.cutreeStatic to identify any outliers. No abnormal samples were detected, resulting in the inclusion of all 109 NASH samples and their clinical grouping information in the Weighted Gene Co-expression Network Analysis (WGCNA) (Fig. 2A). Based on the adjacency matrix and topological overlap matrix, a soft threshold of β = 4 (scale-free R^2^ = 0.91) was selected for subsequent adjacency calculations (Fig. 2B). Genes were grouped into modules, and those with pairwise correlations greater than 0.75 were merged, resulting in a total of 10 modules (Figs. 2C-D, Table S2). Among these, the brown module (*p* = 0.01; R^2^ = − 0.27), green module (*p* = 1e–10; R^2^ = 0.57), magenta module (*p* = 0.04; R^2^ = − 0.2), and blue module (*p* = 0.03; R^2^ = − 0.21) demonstrated significant correlations with NASH fibrosis (Fig. 2E). The Gene Significance within these modules was also statistically significant for the brown, green, magenta, and blue modules (Fig. 2F). Additionally, the Module Significance for the green module was notable (Figure S1). By intersecting genes from these four key modules with the previously identified 104 differentially expressed genes (DEGs), we narrowed down to 74 critical genes associated with NASH fibrosis (Fig. 2G).

To further investigate the primary functions, pathways, and underlying mechanisms linked to NASH fibrosis, we performed functional enrichment analysis. KEGG pathway analysis revealed significant enrichment in pathways such as focal adhesion, PI3K-Akt signaling, and ECM-receptor interaction (Fig. 2H), highlighting essential intercellular and intracellular signaling processes involved in NASH fibrosis. Additionally, GO enrichment analysis identified significant changes in molecular functions (MF), cellular components (CC), and biological processes (BP) related to fibrosis. Notably, fibrosis-related biological processes like extracellular matrix organization, extracellular structure organization, and collagen fibril organization were enriched, along with cellular components such as collagen-containing extracellular matrix, collagen trimers, and collagen trimer complexes. Furthermore, fibrosis-associated molecular functions, including extracellular matrix structural components and those providing tensile strength, were highlighted (Figs. 2I–J). These findings affirm the reliability of the identified gene set. Additionally, we uncovered novel functions not previously linked to NASH fibrosis, such as cellular responses to amino acid stimuli and ligand-gated monoatomic cation channel activity (Figs. 2I–J), offering new theoretical insights for future research in these emerging areas.

### Construction and establishment of NASH fibrosis diagnostic model

**Fig. 3:**
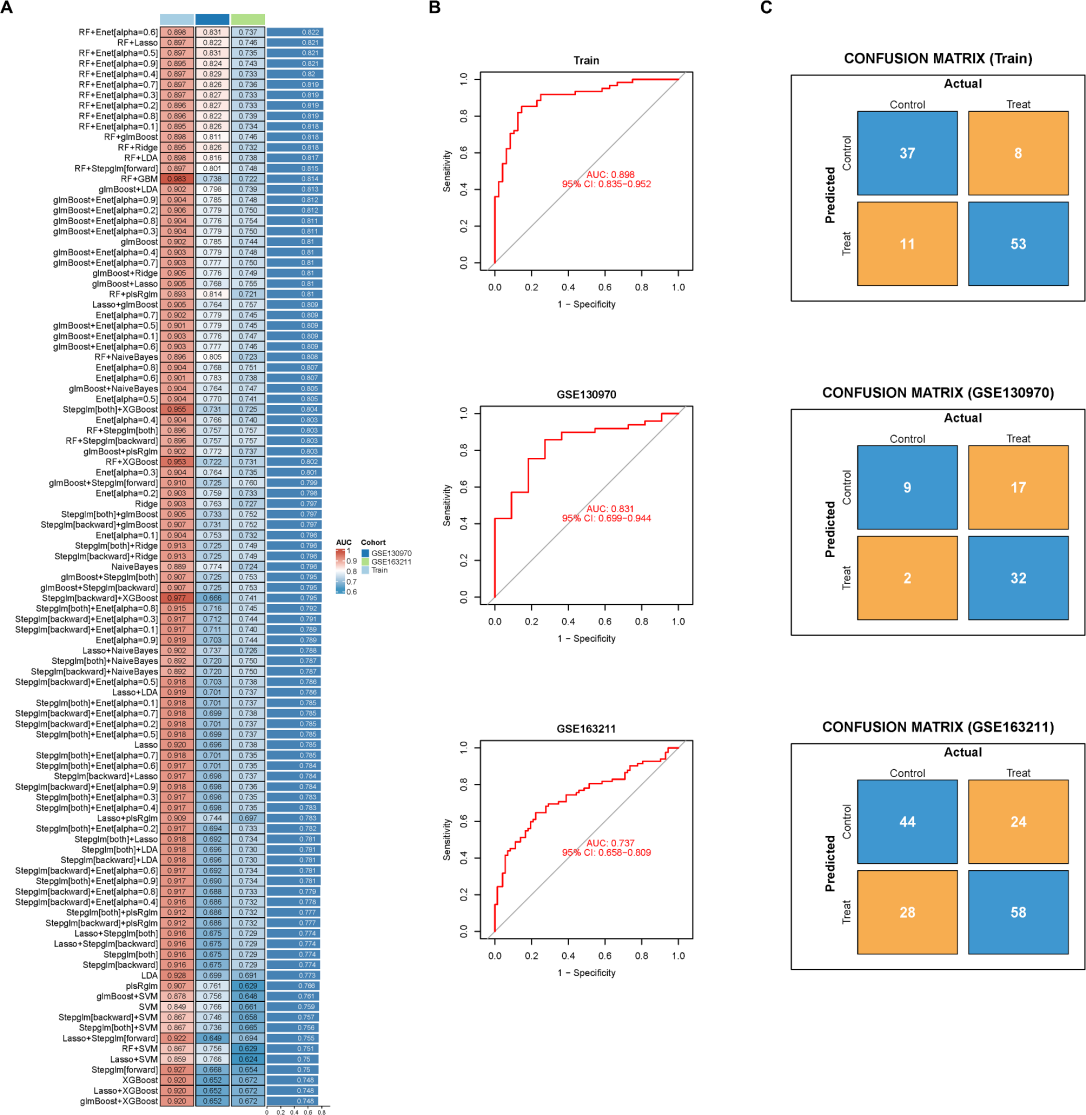
Construction of NASH fibrosis diagnosis model based on machine learning model. (**A**) The heatmap illustrates the area under the curve (AUC) of the performance metrics for both the training and validation sets, evaluated across 12 machine learning models and 103 algorithms. The variable selection and model-building algorithms were used in the order shown in the text. (**B**) The ROC curves demonstrate the testing performance of the model based on the RF (Random Forest) + Enet (Elastic Net, α = 0.6) algorithm for both the training and validation sets. (**C**) The confusion matrix reveals the sensitivity and specificity of the diagnostic model in detecting NASH fibrosis within the training and validation sets.

Based on the expression profiles of key genes involved in NASH fibrosis, we employed a machine learning integration approach to develop a robust diagnostic model for NASH fibrosis. This model was validated using two external datasets, GSE130970 and GSE163211. Using a leave-one-out cross-validation (LOOCV) framework, we fitted 103 different diagnostic models and calculated the C-index for each model across all validation datasets (Fig. 3A, Table S3). Notably, all 103 machine learning models demonstrated strong performance (with mean AUC values exceeding 0.7), affirming the validity of the key genes identified for NASH fibrosis. Among them, the RF + Enet[alpha = 0.6] model was the most effective, achieving a mean AUC of 0.822 and incorporating five key genes: LUM, COL1A2, THBS2, COL5A2, and NTS (Fig. 3A, Table S4). This model exhibited strong diagnostic capabilities in both the training set and the external validation sets (GSE130970 and GSE163211), with AUC values of 0.898, 0.831, and 0.737, respectively (Fig. 3B). Additionally, we visualized the confusion matrix to assess the diagnostic accuracy of the model, which showed excellent specificity and sensitivity in both the training and validation datasets (Fig. 3C).

### Expression patterns and interactions of genes in the NASH fibrosis diagnostic model

**Fig. 4:**
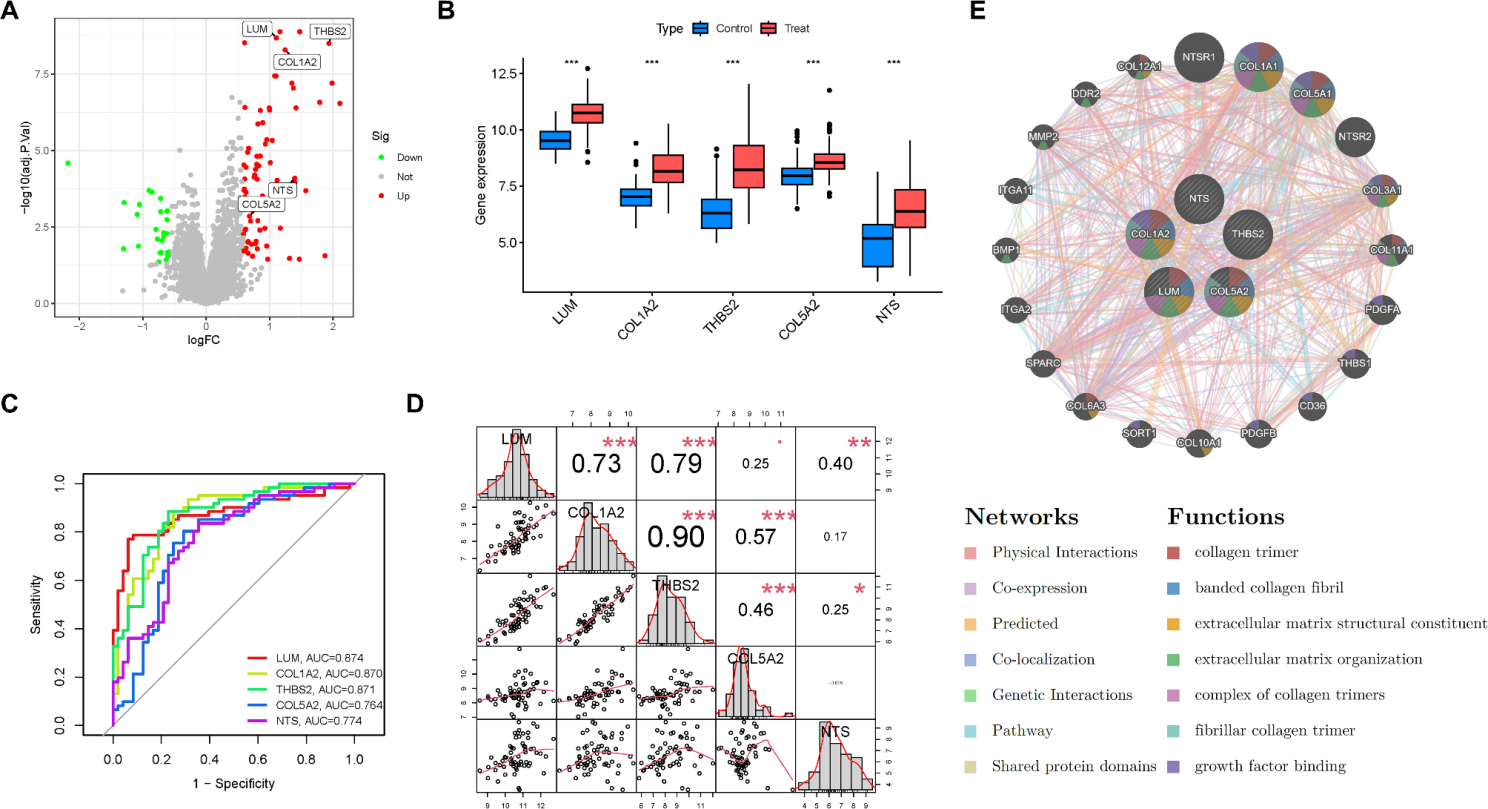
Characterization of key genes in NASH fibrosis in diagnostic models. (**A**) The volcano plot illustrates the differential expression and statistical significance of key genes in the diagnostic model related to NASH fibrosis. (**B**) The box plot displays the relative expression levels of key genes in various diagnostic models for NASH fibrosis. (**C**) The ROC curves demonstrate the diagnostic efficacy of different key genes in the diagnostic models for NASH fibrosis. (**D**) The Pearson correlation analysis reveals the relationships between key genes in the diagnostic model, assessing their synergistic or antagonistic interactions. (**E**) Based on GeneMANIA (http://genemania.org/), this analysis explores the interactions and functional predictions of key genes in the diagnostic model. All images were created using R software (version 4.3.1, https://www.r-project.org).

Next, we illustrated the differential expression distribution of the five model genes in NASH fibrosis using a volcano plot (Fig. 4A). Interestingly, all five model genes are highly expressed in NASH fibrosis, indicating that they are potential risk factors for the condition (Fig. 4B). Additionally, ROC curve analysis demonstrated that these five model genes are effective in diagnosing NASH fibrosis, with LUM showing the highest AUC of 0.874 (Fig. 4C). We also performed Pearson correlation analysis to evaluate the relationships between the model genes, revealing that LUM and THBS2 exhibit strong correlations with all other genes, suggesting they may be hub genes. Notably, LUM, COL1A2, and THBS2 show the most significant correlations among them (Fig. 4D). Furthermore, using GeneMANIA (http://genemania.org/), a tool designed for exploring gene interactions and functional predictions, we found that the model genes collectively participate in and regulate critical NASH fibrosis functions and pathways, including collagen trimer formation, banded collagen fibril assembly, and extracellular matrix structural components (Fig. 4E). These findings further validate the reliability of our diagnostic model.

### Functional enrichment analysis highlighted the primary roles of each model gene in the NASH fibrosis process

**Fig. 5:**
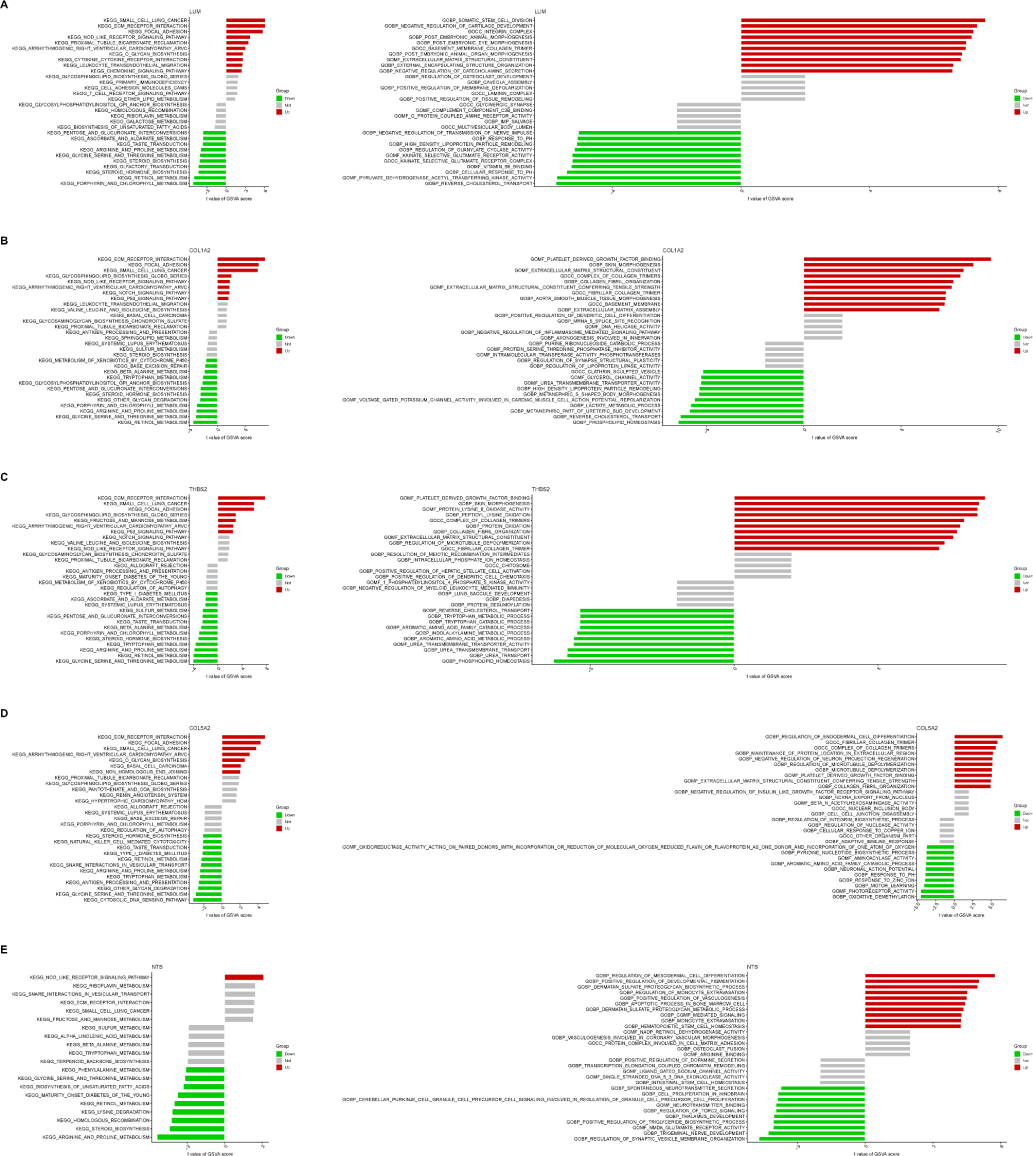
GSVA functional enrichment analysis reveals the unique roles of genes in different diagnostic models in NASH fibrosis. (**A**) GSVA analysis of NASH samples with varying LUM expression levels was conducted using KEGG and GO databases. (**B**) GSVA analysis of NASH samples with different COL1A2 expression levels was performed utilizing KEGG and GO databases. (**C**) KEGG and GO databases were used for GSVA analysis of NASH samples with different THBS2 expression levels. (**D**) GSVA analysis was carried out on NASH samples with varying COL5A2 expression levels, based on KEGG and GO databases. (**E**) GSVA analysis of NASH samples with different NTS expression levels was conducted using KEGG and GO databases.

Gene Set Variation Analysis (GSVA) is a non-parametric, unsupervised approach for assessing gene set enrichment across microarray or transcriptomic data. It transforms sample-specific gene expression matrices into matrices of gene set expression levels, enabling the evaluation of differential metabolic pathway enrichment across samples. Our GSVA results suggest that LUM predominantly influences pathways like KEGG ECM-receptor interaction and GOCC integrin complex, indicating its role in promoting NASH fibrosis through the regulation of inflammatory responses (Fig. 5A). COL1A2 is mainly linked to KEGG focal adhesion and GOCC collagen trimer complex, implying its involvement in directly driving fibrosis by enhancing fibrin production in NASH fibrosis (Fig. 5B). THBS2 and COL5A2 display enrichment patterns similar to COL1A2 (Figs. 5C–D). NTS, on the other hand, shows unique functions related to NASH fibrosis regulation, such as involvement in the GG NOD-like receptor signaling pathway, KEGG riboflavin metabolism, and GOBP regulation of mesodermal cell differentiation, pointing to its potential fibrogenic roles in cellular processes (Fig. 5E).

Gene Set Enrichment Analysis (GSEA) is a method for evaluating the distribution patterns of predefined gene sets within a ranked gene expression profile based on phenotype relevance. By comparing the expression levels of genes within these sets to those across the entire genome, GSEA assesses their contributions to specific phenotypes. In addition, GSEA can be cross-correlated with GSVA to demonstrate the accuracy of our analysis results. Our GSEA revealed that the model genes are associated with key fibrosis-related pathways, including KEGG ECM-receptor interaction and KEGG cytokine-cytokine receptor interaction, which is consistent with the GSVA analysis. Notably, each gene is also linked to distinct pathways that merit further exploration (Figure S2A–E).

### Changes in the immune microenvironment of NASH fibrosis

**Fig. 6:**
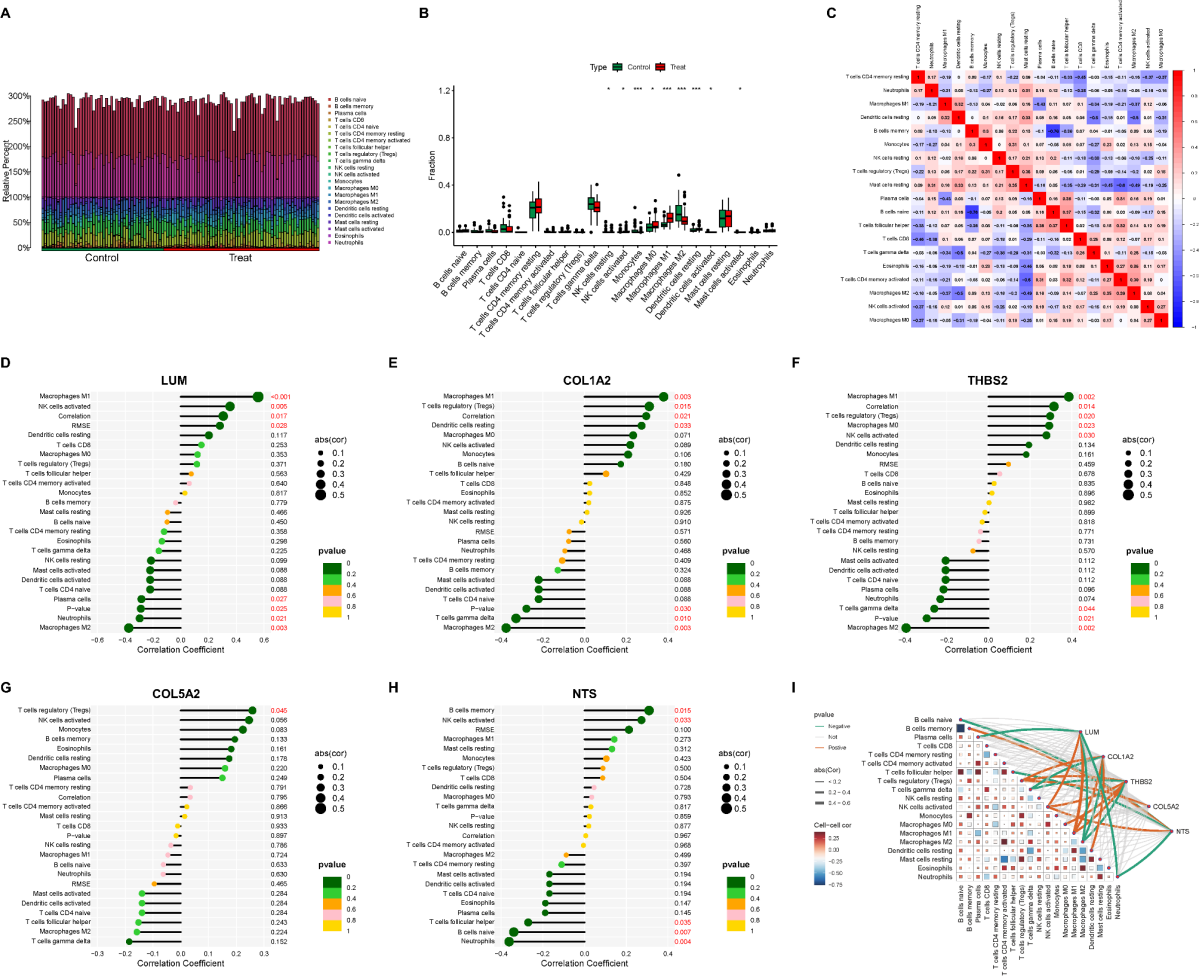
Immune infiltration analysis reveals the immune microenvironment status in NASH fibrosis. (**A**) The bar chart illustrates the percentage distribution of various immune cells or immune functions within NASH samples. (**B**) The bar chart displays the abundance of different immune cells or immune functions across different NASH subgroups. (**C**) The correlation heatmap shows the relationships between various immune cells or immune functions. (**D**) The dendrogram reveals the correlation between LUM expression levels and immune cells or immune functions. (**E**) The dendrogram illustrates the correlation between COL1A2 expression levels and immune cells or immune functions. (**F**) The dendrogram depicts the correlation between THBS2 expression levels and immune cells or immune functions. (**G**) The dendrogram presents the correlation between COL5A2 expression levels and immune cells or immune functions. (**H**) The dendrogram demonstrates the correlation between NTS expression levels and immune cells or immune functions. (**I**) The correlation heatmap displays the regulatory effects of different key genes in diagnostic models on cell communication in NASH fibrosis. All images were created using R software (version 4.3.1, https://www.r-project.org). Statistical significance is denoted as *p < 0.05, **p < 0.01, ***p < 0.001.

Research indicates that NASH (Non-Alcoholic Steatohepatitis) fibrosis may be linked to immune regulation^14,15^. To investigate this, we employed the CIBERSORT algorithm to analyze the immune cell composition and immune function states in NASH samples, aiming to understand the immune microenvironment in NASH fibrosis. Our findings reveal a reduction in resting NK cells, alongside a significant increase in activated NK cells and monocytes, as well as an elevation in pro-inflammatory M1 macrophages. Conversely, alternatively activated M2 macrophages, typically involved in wound healing, showed a marked decline (Figs. 6A–B). These findings suggest that immune cell-mediated inflammation is substantially heightened in NASH fibrosis and could play a critical role in driving fibrosis progression.

Additionally, we examined the interactions between different immune cell types in NASH fibrosis to discern their cooperative or antagonistic roles during disease progression. The analysis revealed that resting CD4 memory T cells and resting mast cells are negatively correlated with activated NK cells and other inflammatory responses (Fig. 6C), indicating a potential protective function in NASH fibrosis. In contrast, follicular helper T cells and eosinophils showed positive correlations with activated NK cells and inflammation (Fig. 6C), suggesting they may exacerbate the inflammatory environment in NASH fibrosis. Correlation analysis between each model gene and immune cell types or functional states further revealed key insights. Notably, LUM, COL1A2, and THBS2 were positively correlated with M1 macrophages and negatively with M2 macrophages, indicating these genes primarily influence inflammatory responses in NASH (Figs. 6D–F). COL5A2 showed a unique positive association with regulatory T cells (Tregs) (Fig. 6G), while NTS was linked to increased memory B cell activity and decreased neutrophil activity (Fig. 6H). A correlation heatmap provided additional clarity on the intercellular communication modulated by each model gene. LUM primarily negatively regulated interactions between plasma cells and naive B cells (Fig. 6I), while COL1A2 positively regulated interactions between plasma cells and gamma delta T cells (Fig. 6I). Both THBS2 and COL5A2 positively influenced the interaction between M0 macrophages and activated NK cells (Fig. 6I), whereas NTS mainly negatively regulated interactions between Tregs and memory B cells (Fig. 6I).

## Discussion

In this study, we integrated and standardized datasets related to NASH and NASH-related fibrosis (GSE48452, GSE49541, and GSE89632) to identify critical gene clusters involved in the progression of NASH fibrosis through differential analysis and WGCNA. Using these genes, we constructed diagnostic models for NASH fibrosis employing 103 machine learning algorithms and validated the models with two external datasets (GSE130970 and GSE163211). Our results demonstrated that all machine learning models based on our gene set performed well (with mean AUC values above 0.7), with the RF + Enet[alpha = 0.6] model showing the best performance (mean AUC of 0.822). This model was comprised of five key genes: LUM, COL1A2, THBS2, COL5A2, and NTS. Gene function enrichment analyses, including KEGG, GO, GESA, and GSVA, revealed that these five key genes play significant roles in pathways such as collagen trimer, banded collagen fibril, and extracellular matrix structural constituent, suggesting their involvement in the potential mechanisms underlying the progression from NASH to liver fibrosis. Additionally, immune cell infiltration analysis indicated that cells like Macrophages M1 might be actively involved in the NASH fibrosis process. Our findings highlight critical factors in the progression of NASH to liver fibrosis and provide a robust diagnostic model for assessing fibrosis risk in NASH patients, facilitating early intervention. Furthermore, the unique immune microenvironment associated with NASH fibrosis suggests potential for immune-based therapies. Our research offers a solid theoretical foundation for the clinical management of NASH and the development of future targeted treatments.

NASH (Non-Alcoholic Steatohepatitis) fibrosis significantly contributes to increased mortality and reduced quality of life in patients. Although liver biopsy remains the gold standard for diagnosing NASH-related fibrosis, its invasive nature, associated risks, and high cost limit its practicality in clinical settings. As obesity and metabolic syndrome become more prevalent, NASH increasingly burdens healthcare systems and the economy^16^. Therefore, developing simpler and more efficient screening methods for NASH is of great importance^17^. In response to this challenge, we employed 103 different machine learning algorithms to create a model for identifying individuals at high risk of NASH fibrosis. These models performed well, with mean AUC values consistently above 0.7, underscoring the relevance of the key genes identified for NASH fibrosis. Notably, the RF + Enet[alpha = 0.6] model emerged as the top performer, with a mean AUC of 0.822, integrating five key genes: LUM, COL1A2, THBS2, COL5A2, and NTS. This model demonstrated strong diagnostic performance across both the training set and external validation datasets GSE130970 and GSE163211, achieving AUCs of 0.898, 0.831, and 0.737, respectively. Additionally, the confusion matrix visualized the model’s diagnostic accuracy, showcasing high specificity and sensitivity in both the training and validation sets. This diagnostic model holds promise in aiding clinical decision-making, offering patients more cost-effective and accurate diagnostic and therapeutic options.

NASH is a complex, multifactorial disease with an unclear etiology. Typically, it involves a combination of factors, such as steatosis, liver damage, and inflammation, which collectively can lead to fibrosis and even hepatocellular carcinoma (HCC) in some cases^18^. Although NASH generally develops from NAFLD, not all cases progress to fibrosis. Additional stressors, including lipotoxicity, oxidative stress, and inflammation, drive NASH fibrosis by activating cellular stress pathways, leading to hepatocyte death, inflammation, and fibrotic changes^19^. Through gene function and pathway enrichment analyses, we identified several known fibrosis-related pathways in NASH, such as KEGG’s ECM RECEPTOR INTERACTION and GOCC’s INTEGRIN COMPLEX. Interestingly, we also uncovered novel pathways not previously linked to NASH fibrosis, including GG’s NOD-LIKE RECEPTOR SIGNALING PATHWAY, KEGG’s RIBOFLAVIN METABOLISM, and GO’s REGULATION OF MESODERMAL CELL DIFFERENTIATION. These newly identified pathways may have significant roles in NASH fibrosis and warrant further investigation.

The liver is not only vital for metabolism and detoxification but also plays a crucial role in immunity, housing a diverse array of innate and adaptive immune cells^14^. Its highly vascularized architecture, with fenestrated capillaries called liver sinusoids, creates a unique environment where immune cells are exposed to pathogens from both the blood and the gut^14^. In the context of NASH, the immune cell landscape in the liver undergoes significant changes, leading to uncontrolled inflammation, hepatocyte death, and fibrosis, all of which exacerbate the disease^20^. Key immune cells involved in this process include innate-like T cells, conventional CD8 + and CD4 + T cells, and neutrophils. Neutrophil accumulation is an early event in NASH, promoting inflammation and liver injury, particularly through the release of neutrophil extracellular traps (NETs) or NETosis^4^. Additionally, dendritic cells (DCs), particularly type 1 conventional dendritic cells (cDC1), increase in number and contribute to liver inflammation, potentially activating CD8 + T cells and worsening liver damage. Monocytes are also rapidly recruited to the liver, where they can differentiate into pro-inflammatory M1 macrophages or generate monocyte-derived Kupffer cells^21^. While most research on immune cells in NAFLD progression has been conducted using murine models, our study confirms that NASH fibrosis is associated with a significant reduction in resting NK cells and an increase in activated NK cells and monocytes. Additionally, there is a marked rise in pro-inflammatory M1 macrophages and a significant decline in alternatively activated M2 macrophages, which are involved in wound healing. These findings suggest that immune cell-driven inflammation plays a critical role in NASH and may significantly contribute to fibrosis progression. We also identified five key genes that are integral to immune cell communication, and their underlying mechanisms should be further explored in future studies. Given the current lack of effective therapies to mitigate liver fibrosis, there is a pressing need for innovative approaches. Recent studies have highlighted the potential of immunotherapy in reducing extracellular matrix deposition in NASH mouse models^22^. The key immune-regulatory genes identified in our study may serve as promising therapeutic targets in NASH fibrosis.

NAFLD (Non-Alcoholic Fatty Liver Disease) is a complex disorder, with NASH representing a pivotal stage that leads to significant liver damage and a high risk of progressing to fibrosis. Although NASH develops in the context of metabolic changes, it also has a substantial immune-inflammatory component^23^. During NASH, a vast network of immune cells is mobilized, and our research offers a comprehensive view of how specific immune cell subpopulations contribute to fibrosis in NASH. Additionally, we examined the interactions between various immune cell types and between immune cells and matrix cells, providing new insights into the complexity of NASH^24^.

## Conclusion

Our research has developed a reliable diagnostic model for NASH fibrosis, leveraging 103 different machine learning algorithms. This model is designed to either diagnose NASH fibrosis or assess the risk of liver fibrosis progression in NASH patients. Additionally, we have identified critical changes in signaling pathways and functional states associated with NASH fibrosis, providing a solid theoretical foundation for further mechanistic studies. This study is subject to certain limitations due to the absence of datasets for NASH and NASH-related fibrosis from Asian populations, which may render our model more applicable to Western populations. Consequently, we anticipate the collection of NASH and NASH-related fibrosis samples from Asian cohorts in the future. Following extensive transcriptomic sequencing, we aim to further validate the model to assess its generalizability.

## Electronic supplementary material

Below is the link to the electronic supplementary material.

Supplementary Material 1.

Supplementary Material 2.

Supplementary Material 3.

Supplementary Material 4.

Supplementary Material 5.

## Data Availability

The results published here are in whole based upon data generated by the GEO datasets: https://www.ncbi.nlm.nih.gov/geo/. All other relevant data can be found in the supplementary material and will be made available on request.
