# Supplementary material for: Machine learning-based integration reveals reliable biomarkers and potential mechanisms of NASH progression to fibrosis
Source: Sci Rep. 2025 Apr 11;15:12411. doi: 10.1038/s41598-025-97670-4 (PMC11992153; doi:10.1038/s41598-025-97670-4)
Supplement: Supplementary file 5 — Supplementary Material 5. [file 41598_2025_97670_MOESM5_ESM.docx]

**Figure S1 Further analysis of key modules based on WGCNA analysis**

A) Scatter plot of MM vs. GS for each module of NASH fibrosis.

**p* < 0.05, ***p* < 0.01, ****p* < 0.001.





**Figure S2 Functional enrichment analysis of different model genes in NASH fibrosis based on GSEA**

A) GSEA analysis of NASH samples with different LUM expression levels was performed using KEGG and GO databases.

B) GSEA analysis of NASH samples with varying COL1A2 expression levels was conducted utilizing KEGG and GO databases.

C) GSEA analysis based on KEGG and GO databases was carried out for NASH samples with different THBS2 expression levels.

D) GSEA analysis for NASH samples with varying COL5A2 expression levels was performed using KEGG and GO databases.

E) GSEA analysis of NASH samples with different NTS expression levels was conducted using KEGG and GO databases.
